# Hyaluronan as an immunological adjuvant: a novel application for an ancient molecule

**DOI:** 10.1038/s41423-023-01024-x

**Published:** 2023-05-17

**Authors:** D. Carpanese, A. Dalla Pietà, A. Rosato

**Affiliations:** 1 Veneto Institute of Oncology IOV-IRCCS, Padova, Italy; 2 University of Padua, Department of Surgery, Oncology and Gastroenterology, Padova, Italy

**Keywords:** Adjuvants, Protein vaccines

Vaccines are among the most important scientific breakthroughs in human history. However, both the recent COVID-19 pandemic and cancer prevalence highlighted the requirements for new vaccination platforms that are more effective in stimulating complex immune responses, including the activation of CD4^+^ and CD8^+^ T-cell-mediated immunity and the induction of immunological memory. In this regard, protein-/peptide-based vaccines represent valuable options that are based on a balance between a good safety profile and a relatively rapid manufacturing time; nonetheless, efficient adjuvants are needed for protein-based vaccines to overcome weak immunogenicity. Toll-like receptor (TLR) agonists have been advanced as promising adjuvants for the development of protein-based vaccines that can overcome all the shortcomings of clinically available formulations, as these agonists fine-tune the crosstalk between innate and adaptive immune systems. However, a critical challenge remains the identification of TLR-agonist adjuvants with increased potency but minimal toxicity. In this context, natural polymers (NPs) and their derivatives originating from plants, animals, or microbes are good candidates for use in the creation of vaccines with both high safety and efficacy profiles [1].

Since 1880, hyaluronan (HA) has been the most extensively studied and ubiquitous NP in the extracellular matrix. In addition to its simple structure, this linear glycosaminoglycan is endowed with remarkable physicochemical properties such as biodegradability, biocompatibility, and nontoxicity, leading to high versatility that has been successfully exploited for use in several medical applications. HA is characterized by a very rapid turnover, and HA homeostasis in tissue is regulated by the fine-tuned balance between synthesis and degradation that plays a key role in determining not only the amount of HA in the human body but also the HA molecular weight (MW). The latter, in turn, is closely related to the biological functions of HA under both physiological and pathological conditions. Indeed, HA fragments with different MWs exhibit diverse and sometimes opposite biological functions, such as promoting or blocking inflammation, angiogenesis, malignant transformation and immune surveillance [2], mainly by binding to a plethora of receptors/proteins. Notably, an important aspect of HA signaling is based on its interaction with TLRs. Indeed, low-molecular-weight (LMW) HA (100-500 kDa) has been shown to engage a receptor complex comprising CD44 and TLRs, inducing cytokine release [3], and HA oligomers (with fewer than 10 HA disaccharides) have been reported to function as damage-associated molecular pattern (DAMP) molecules by specifically binding TLR2 and TLR4 [4].

**Fig. 1:**
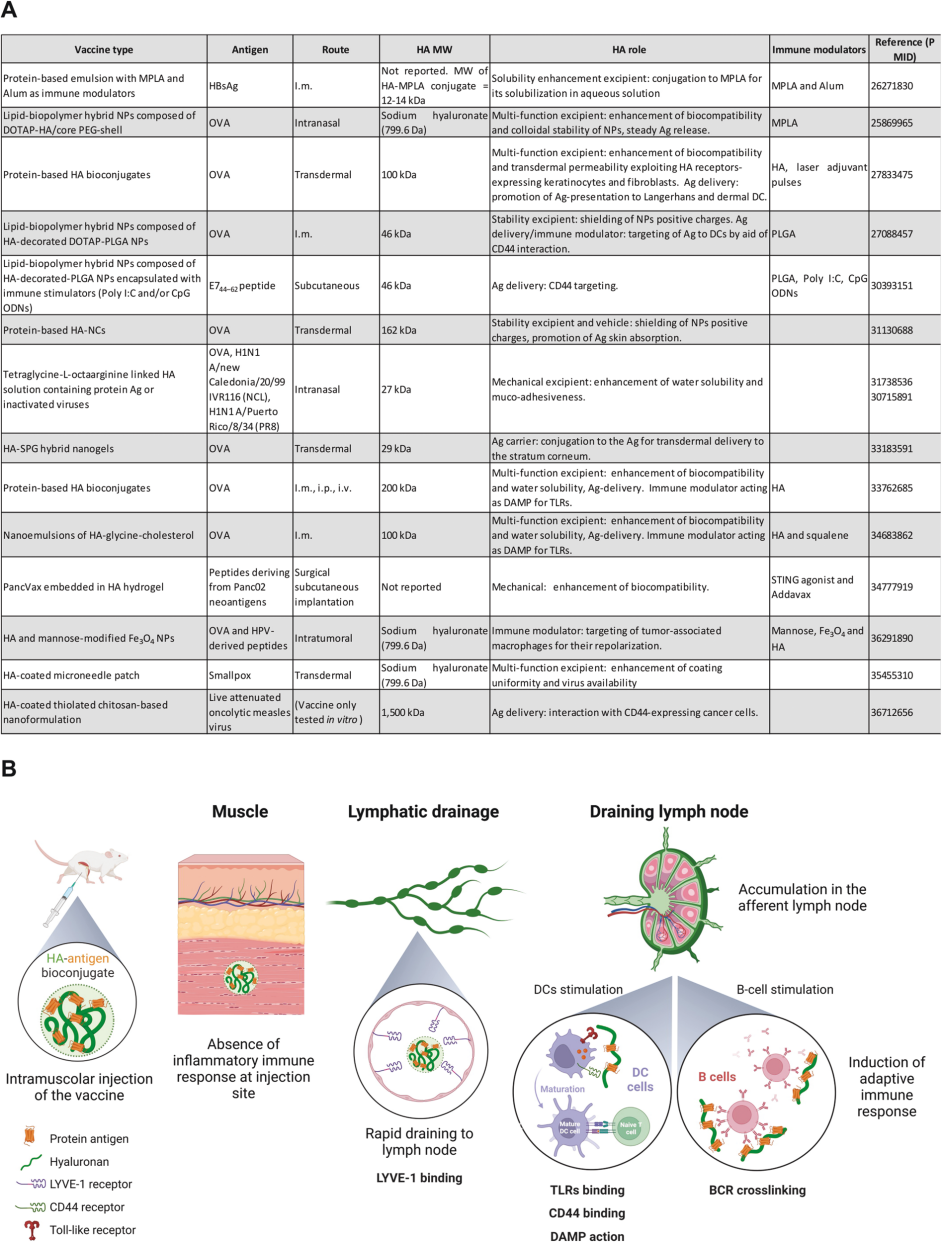
**A** The table summarizes HA-based vaccines validated preclinically from 2015 to 2023. MPLA = the detoxified derivative of lipopolysaccharide (LPS) isolated from the gram-negative *Salmonella Minnesota* R595 bacterial strain; HBsAg = recombinant hepatitis B surface antigen; NPs = nanoparticles; DOTAP = 1,2-dioleoyl-3-trimethylammonium-propane; PEG = poly(ethylene glycol); poly I:C = polyinosinic-polycytidylic acid; CpG ODN = CpG oligodeoxynucleotides; NCs = nanocapsules; SBG = β-glucan schizophyllan; STING = stimulator of interferon genes; HPV = human papilloma virus. **B** Graphical summary of the HA mechanism of action. Following intramuscular administration, HA-bioconjugates rapidly enter draining lymph nodes due to their small size and ability to bind the LYVE-1 receptor without inducing any inflammatory response at the injection site. In the draining lymph nodes, the bioconjugate is retained longer than soluble antigens, and HA binding to TLRs and CD44 allows optimal interactions with APCs, inducing the maturation of these cells. Additionally, the repetitive display of multiple antigens bound to an HA chain facilitates the crosslinking of B-cell receptors (BCRs) and results in sustained activation of B cells. *Created with BioRender.com*

Despite its close connection with the immune system, this polymer has been used mostly as a drug carrier [5], and in vaccinology, it has been exploited merely as an excipient, because of its physicochemical properties, or as a delivery vehicle, but its inherent potential as a direct immunostimulant has never been leveraged (Fig. 1A). In a paper, Kim et al. [6] reported that HA used as a “nanocarrier” conjugated to protein antigens (Ags) increased ovalbumin (OVA) immunogenicity in the context of transdermal vaccination that also required a “laser adjuvant”. This approach was laborious and largely unadaptable, and therefore, its potential application to massive vaccination campaigns (e.g., COVID-19) has not been considered, and no articles reported thus far have suggested using HA as an adjuvant per se.

However, we recently reported clear evidence showing that HA fragments of approximately 200 kDa exhibited intrinsic adjuvant properties when covalently conjugated to an Ag [7], leading to vaccine prototypes that were highly efficient in increasing protein immunogenicity without the need for any other adjuvant compound; moreover, these vaccine prototypes showed a reduced need for boosters and was effective at low antigen doses. In addition to these beneficial effects, HA induced no local or systemic toxicity, and therefore, HA appears to be an ideal adjuvant that can be used to overcome the limitations of other compounds that present safety drawbacks.

Chemical conjugation to Ag is indispensable for realizing the adjuvant effect of HA, as an injection of a simple mixture of HA and an antigen was not effective in stimulating Ag-specific immune responses [7]. Although the need for a conjugation procedure might be regarded as a limitation, especially from the manufacturing point of view, we speculate that it is very likely also the key to the success of the HA-based vaccination approach. Typically, polymer conjugates are developed to improve the tolerability and half-life of peptides/proteins and drugs with poor systemic pharmacokinetics. Additionally, conjugation of TLRs to antigens has been widely described as a critical requirement for successful agent delivery, resulting in increased antigen presentation and processing efficiency by antigen-presenting cells (APCs) [8]. Therefore, one of the most important factors for the stimulation of an immune response is efficient trafficking of an Ag to the lymph node, which, in turn, depends on the size of the transported molecule [9]. Therefore, fine-tuning vaccine particle size is a fundamental process in the development of novel vaccines. In our system, the range of molecule sizes for HA effectiveness is quite narrow and therefore likely plays an important role in the mechanism of action of the bioconjugate. Indeed, since a size from 5 to 50 nm was the optimal dimension range for passive trafficking of HA into lymphatic vessels [9], an 200 kDa HA-protein bioconjugates with a diameter of approximately 15 nm may be optimal to assure prompt delivery of high levels of antigen to lymph nodes, as well as for its interaction with APCs. In contrast, neither a 500 kDa nor a 15 kDa HA-based bioconjugate showed an adjuvant effect, which is likely explained by the different biological fates after injection in vivo. In fact, the 15-kDa HA-conjugate very rapidly drained from the injection site into the lymphatic system, moving through through lymph nodes and finally entering the blood flow, without interacting with APCs in the lymph nodes. Moreover, the movement of the 500-kDa moiety is likely inhibited at the injection site and was locally degraded into polymers of different MWs, which made them less likely to adhere to lymph nodes. Overall, our data delineate a situation where HA-antigen bioconjugates are essentially inert and nonreactogenic at the site of injection, with Ag capture and presentation taking place in draining lymph nodes (Fig. 1B). In addition to the passive flow rate of HA-conjugates based on size, we speculate that the HA-antigen bioconjugates are also transported to lymph nodes after the HA moiety binds to the receptor LYVE-1, which is expressed in the lymphatic system.

Once in a lymph node, an HA-conjugated antigen can be efficiently captured by DCs and macrophages, contributing to their maturation/activation by binding to CD44 and TLRs, ultimately leading to efficient stimulation of the T-cell compartment. Furthermore, in B cells, the multimeric nature of HA-protein bioconjugates likely triggers more effective and sustained B-cell receptor (BCR) cross-linking and signaling than is induced by a single antigen moiety, while assuring costimulation through TLRs interacting with the polymer component [10].

Although additional work is certainly required to clearly elucidate the mechanism of HA action as an adjuvant, the versatility of the HA-based vaccination approach has been supported by recent data. A COVID-19 prototype vaccine based on the conjugation of HA to the receptor-binding domain (RBD) of the SARS-CoV-2 spike protein induced a markedly high level of neutralizing antibodies against different viral variants and elicited long-lasting immunity capable of fully protecting infected mice from lethality. In the cancer setting, a conjugate composed of HA linked to the extracellular domain of the HER2 receptor efficiently constrained the growth of HER2 + breast tumors and increased survival of mouse models with different types of breast cancer.

Overall, we strongly believe that the potential for HA use in vaccinology is largely underestimated and that its inherent adjuvanticity can lead to the design of an entire new class of vaccines endowed with increased efficacy and safety.
